# Supplementary material for: Phase-Inverted Copolymer Membrane for the Enhancement of Textile Supercapacitors
Source: Polymers (Basel). 2022 Aug 19;14(16):3399. doi: 10.3390/polym14163399 (PMC9415922; doi:10.3390/polym14163399)
Supplement: Supplementary file 1 [file polymers-14-03399-s001.zip › polymers-1861837-supplementary.pdf]

Support information for

Phase inverted co-polymer membrane for the enhancement of textile supercapacitors

Sheng Yong, Nicholas Hillier and Stephen Paul Beeby

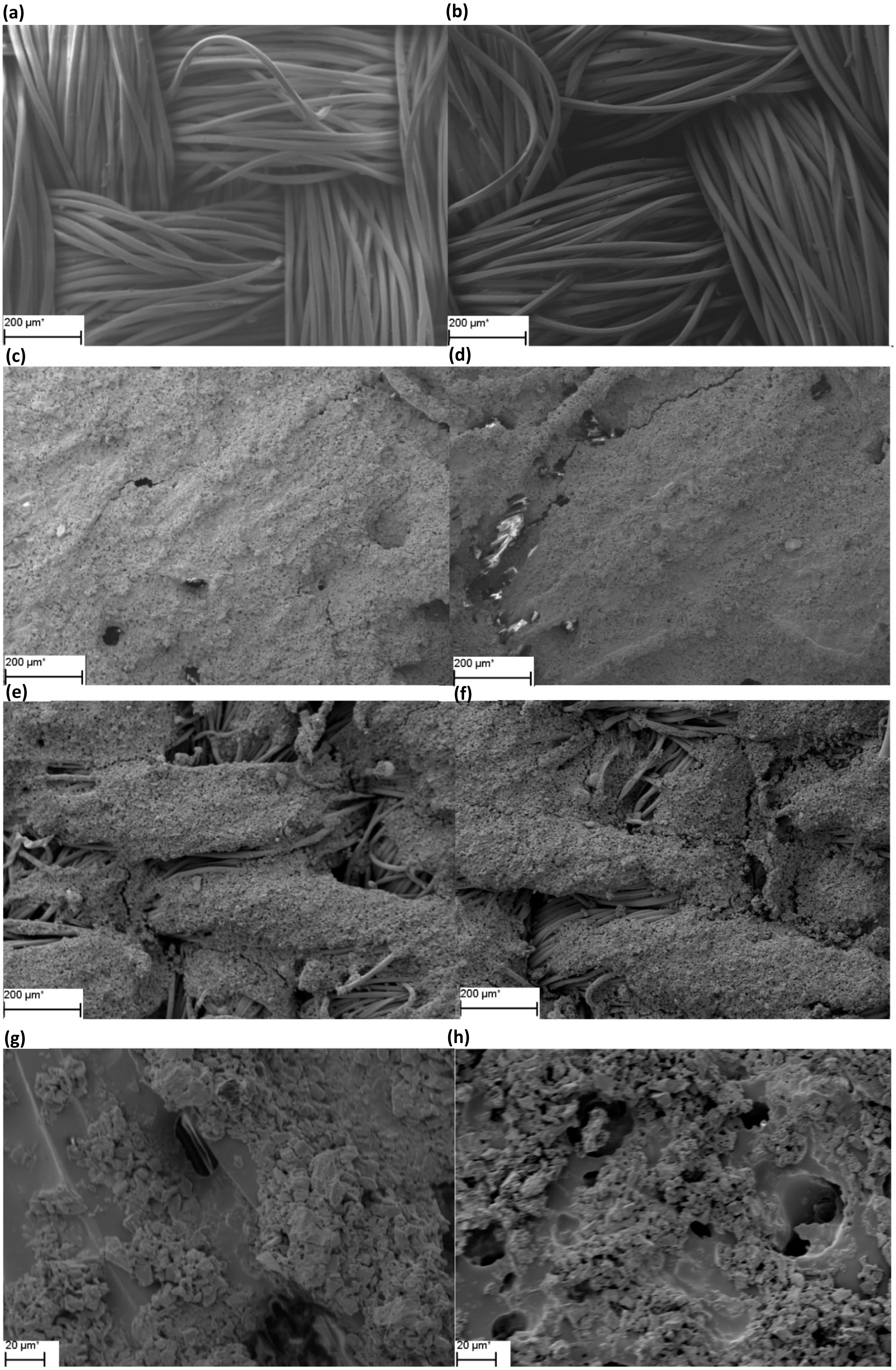
(a)
200 µm*
(b)
200 µm*
(c)
200 µm*
(d)
200 µm*
(e)
200 µm*
(f)
200 µm*
(g)
20 µm*
(h)
20 µm*

Figure S1. SEM photos showing plan views of (a) PC 150 textile, (b) P textile (c) PC 150 textile with membrane and carbon coating, (d) P textile with membrane and carbon coating, (e) PC 150 textile with carbon coating without membrane, (f) P textile with carbon coating without membrane (g) PC 150 textile with membrane and carbon coating (high magnification), (h) P textile with membrane and carbon coating (high magnification)

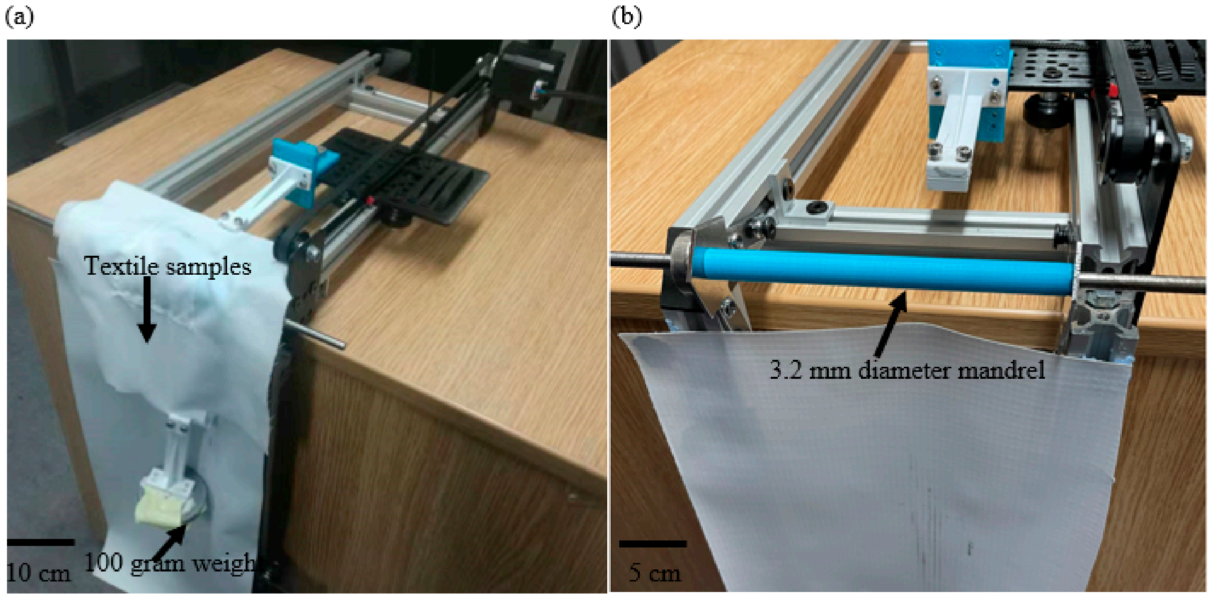

Figure S2 (a) Bending test set up, (b) Mandrel used in bending test
